# Tetrodotoxin for Moderate to Severe Cancer-Related Pain: A Multicentre, Randomized, Double-Blind, Placebo-Controlled, Parallel-Design Trial

**DOI:** 10.1155/2017/7212713

**Published:** 2017-05-07

**Authors:** Neil A. Hagen, Lyne Cantin, John Constant, Tina Haller, Gilbert Blaise, May Ong-Lam, Patrick du Souich, Walter Korz, Bernard Lapointe

**Affiliations:** ^1^Tom Baker Cancer Centre and Division of Palliative Medicine, University of Calgary, 1331 29 Street NW, Calgary, AB, Canada T2N4N2; ^2^ClinForce Services Inc., Vancouver, BC, Canada; ^3^PRA Health Sciences, Victoria, 655 Tyee Rd., Victoria, BC, Canada V9A 6X5; ^4^Statcon Consulting Services, 6 Matson Drive, Caledon, ON, Canada L7E 0A3; ^5^Département d'Anesthésiologie, CHUM, 1560, rue Sherbrooke Est, Pavillon Deschamps, Local FS-1136, Montréal, QC, Canada H2L 4M1; ^6^St. Paul's Hospital, 1081 Burrard Street, Vancouver, BC, Canada V6Z 1Y6; ^7^Département de Pharmacologie et Physiologie, Faculté de Médecine, Université de Montréal, CP 6128 Centre-Ville, Montréal, QC, Canada H3C 3J7; ^8^WEX Pharmaceuticals Inc., 420-1090 West Pender Street, Vancouver, BC, Canada V6E 2N7; ^9^Clinical Research Unit, Jewish General Hospital, Room E-872 3755, Chemin de la Côte Sainte-Catherine, Montréal, QC, Canada H3T 1E2

## Abstract

**Objective:**

This study evaluated subcutaneous injections of tetrodotoxin (TTX) for the treatment of moderate to severe, inadequately controlled cancer-related pain.

**Methods:**

Eligible patients were randomized to receive TTX (30 *μ*g) or placebo subcutaneously twice daily for four consecutive days. Efficacy was assessed using pain and composite endpoints (including pain and quality of life measures), and safety was evaluated using standard measures.

**Results:**

165 patients were enrolled at 19 sites in Canada, Australia, and New Zealand, with 149 patients in the primary analysis “intent-to-treat” population. The primary analysis supports a clinical benefit of TTX over placebo based on the pain endpoint alone with a clinically significant estimated effect size of 16.2% (*p* = 0.0460). The *p* value was nominally statistically significant after prespecified (Bonferroni Holm) adjustment for the two primary endpoints but not at the prespecified two-sided 5% level. The mean duration of analgesic response was 56.7 days (TTX) and 9.9 days (placebo). Most common adverse events were nausea, dizziness, and oral numbness or tingling and were generally mild to moderate and transient.

**Conclusions:**

Although underpowered, this study demonstrates a clinically important analgesic signal. TTX may provide clinically meaningful analgesia for patients who have persistent moderate to severe cancer pain despite best analgesic care. This clinical study is registered with ClinicalTrials.gov (NCT00725114).

## 1. Introduction

Pain related to cancer is highly prevalent. However, existing analgesic treatments do not always alleviate the pain; moreover, medications such as opioids may be inadequately tolerated [1, 2]. Additional analgesic approaches are urgently needed.

Tetrodotoxin (TTX) is a small molecule that blocks voltage-gated sodium channels on neurons. It exerts its analgesic properties by inhibiting the initiation and conduction of impulses in the peripheral nervous system [3, 4]. Clinical trials have been ongoing to evaluate the analgesic effect of TTX in cancer pain [5–8].

There is clinical evidence emerging that the analgesic effect of TTX can last for weeks or even months, after a four-day treatment cycle. The mechanism of action is thought to be primarily due to blockage of one subclass of sodium channels, Na_V_ 1.7, found predominantly on nociceptive neurons in the periphery [3, 4]. However, the mechanism for the prolonged effect demonstrated in many patients is still unclear.

The primary objective of the current study was to compare the efficacy of subcutaneous TTX (trade name Halneuron™) with that of placebo in patients with pain due to advanced cancer or its treatment.

## 2. Methods

This study was a multicentre, randomized, double-blind, placebo-controlled, parallel-design trial of the efficacy and safety of TTX. Men and women over 18 years of age with relatively stable but inadequately controlled moderate to severe cancer-related pain of at least two weeks' duration were recruited. Pain could be somatic, visceral, neuropathic, or mixed and due to cancer or its treatment. Eligible patients were randomized to receive a subcutaneous dose of TTX (30 *μ*g) or an equivalent volume of placebo (1 : 1 ratio) twice daily administered with at least a 6-hour interval between treatment doses, for 4 consecutive days. Patients were followed until pain returned to baseline. Each patient's participation was expected to be at least 3 weeks from start of screening to Day 15 and could last until the end of the analgesic response.

### 2.1. Primary Endpoint

To assure that an analgesic signal could be identified in this complex group of patients, coprimary clinical endpoints were defined [9] that included (1) a composite endpoint (pain plus quality of life (QoL)) and (2) the pain component alone without the QoL component.

The efficacy was evaluated as the proportion of responders who satisfied specified criteria. A responder for the pain endpoint (alone) had to satisfy the following criterion:

1. A ≥30% decrease in mean pain intensity (for worst pain, average pain, or most bothersome pain) or a decrease of ≥50% of opioid equivalent use from baseline, during the early postinjection period (EPIP: Days 5–8) or late postinjection period (LPIP: Days 9–15). During the same period, mean opioid analgesic dose (expressed as morphine equivalents daily dose, MEDD) could not exceed 125% of the mean baseline period use.

A responder for the composite endpoint had to satisfy criterion (1) above, in addition to both of the following criteria:

(2) A ≥30% improvement of QoL in at least 1 descriptor of physical functioning [Brief Pain Inventory (BPI) Q#9A (general activity) or BPI Q#9C (walking ability) or BPI Q#9D (normal work)] during the EPIP or LPIP period.
(3) A ≥30% improvement of QoL in at least 1 descriptor of emotional functioning [BPI Q#9B (mood) or BPI Q#9E (relations with other people) or BPI Q#9G (enjoyment of life)] during the EPIP or LPIP period.

The secondary endpoints were the proportion of responders during the injection period, time to overall response, duration of overall response, time to peak analgesic response, duration of peak analgesic response, pain intensity difference (PID) for worst pain, average pain and most bothersome pain, opioid use (total dose converted to MEDD), and overall or global impression of change (GIC).

The study was also designed to compare the safety of subcutaneous TTX with that of placebo. Safety was evaluated using standard approaches to monitor for and record adverse events (AEs), plus 12-lead ECG, neurological examinations, and routine clinical tests. 

### 2.2. Study Design

Patients were screened for the study and then entered a 4- to 7-day baseline period within 28 days of screening. Following the baseline period, patients were randomized on Day 1 to receive study drug twice daily for 4 consecutive days. Drug administration from Days 1 to 4 was done either in a full-service hospital setting or at a private clinic, depending on the investigator. After the treatment period, all patients were seen again at the facility on Days 5, 8 (EPIP), and 15 (LPIP) for further safety and efficacy evaluations and then every week by telephone or at the clinic, until pain returned to baseline level. The Brief Pain Inventory form and Patient Diary were completed weekly after day 15.

Patients with continued pain relief were assessed beyond 10 weeks. Patients receiving at least 1 but less than 8 doses of study medication (for reasons other than consent withdrawal) were expected to undergo all study procedures per protocol until Day 15 (or until pain returned to baseline level if after Day 15).

The inclusion criteria included ≥18 years of age, a diagnosis of cancer, and stable baseline pain intensity score of ≥4/10 as assessed by numeric rating scale (worst pain in the past 24 hours on the Brief Pain Inventory). Pain stability was defined as a no greater than a 3-point difference between the highest and lowest pain intensity scores (as assessed by BPI Q#5) during the baseline period, and a no more than 50% increase or decrease in opioid analgesic use during the same baseline period without introduction of a new analgesic. Opioid consumption was expressed in MEDD. Patients were allowed one outlier day (pain score or opioid use falling outside the acceptable limit) if, in the opinion of the qualified investigator, there was unusual life circumstance that likely affected the pain experience.

All patients had a history of treatment with analgesics. Patients were maintained on their existing analgesic regimens that included opioids and coanalgesics (antidepressants, anticonvulsants).

The key exclusion criteria included a history of CO_2_ retention or clinically relevant hypoxia, severe renal impairment, prolonged QTc interval, planned initiation of any new anticancer treatment, and female patients with a positive pregnancy test.

### 2.3. Data Analysis

During the conduct of the trial, two interim analyses were undertaken by an arms-length, unblinded Data Monitoring Committee in order to adjust the sample size and make recommendations on early termination. A first interim analysis was conducted after 60 evaluable patients were enrolled and a second after 110 evaluable patients. The statistical analysis included penalties for this approach (O'Brien-Fleming adjustment accounting for two interim analyses).

The Data Monitoring Committee indicated that the first interim analysis showed that the composite endpoint would be futile; however, based on one or more of the single components (pain, QoL emotional functioning, and QoL physical functioning) the results may not be futile. The trial conduct remained unchanged, but following discussion with Canada's drug regulatory agency the decision was made to elevate the importance of the pain intensity reduction portion as a coprimary endpoint, with statistical adjustment to account for possible success on either endpoint.

Assuming a proportion of responders (for each coprimary endpoint) for the placebo group of 20% versus 40% for TTX and adjusting for the 2 coprimary endpoints (using Bonferroni methods), 220 evaluable patients (110 per arm) were required for 80% power and a two-sided test. To obtain 220 evaluable (i.e., per protocol) patients, a total of 254 patients would need to be randomized.

Efficacy and safety analyses were performed on an “intent-to-treat (ITT)” population defined as all randomized patients who had at least 1 injection of study medication and at least 1 postbaseline efficacy assessment. A per-protocol (PP) population of patients without major protocol violations was also prespecified for secondary analyses.

The proportion of responders observed in each treatment arm for the composite endpoint (coprimary #1) and for reduction in pain intensity endpoint (coprimary #2) was summarized and compared using the Wald chi-square test on the (above defined) ITT and per-protocol populations, the ITT population being of primary interest. The Holm-Bonferroni and O'Brien-Fleming methods were used to control for type I error arising from multiple treatment comparisons and for the (multiple) two coprimary endpoints. In addition, trends for differences between treatments were individually tabulated for impact of treatment on pain and QoL (physical and emotional functioning). All hypotheses other than the primary hypothesis tests for the two coprimary endpoints were based on 2-sided tests using a 5% level of significance.

Covariate-adjusted analyses were specified to explore the impact of possible confounders and effect modifiers on the primary results to examine the robustness of the treatment effect across subgroups and for unbiased estimation of treatment effect. The clinically relevant covariables used to assess the robustness of the treatment effect were age, sex, baseline daily opioid use, pain (verbal rating scale), metastases, and site type (outpatient clinic versus inpatient facility).

To address the issue of missing data, for patients who terminated the study prematurely or data missing for other reasons, sensitivity analyses including all 165 randomized patients were conducted using three imputation methods.

The trial protocol underwent formal scientific and ethical review and was approved at each of the participating sites. Full written, informed consent was obtained from each participating subject.

## 3. Results

Patients were enrolled at 19 of 21 participating sites in Canada, Australia, and New Zealand. The study opened in April 2008 and last data was collected in November 2012. 165 patients were randomized, 77 patients (46.7%) in the TTX arm and 88 (53.3%) in the placebo arm. The intent-to-treat (ITT) population consisted of 149 patients, 65 (84.4%) in the TTX group and 84 (95.5%) in placebo. The per-protocol population included 50 patients (64.9%) in the TTX group and 77 patients (87.5%) in placebo. While the intent was to enroll 254 patients, because of associated costs, the sponsor decided to halt the study enrollment at 165 patients. The primary analysis included the 149 completed patients who fulfilled criteria for ITT inclusion (defined above).

All patients had a cancer diagnosis, including a wide range of solid and other tumors. The cohort ranged in age from 25 to 84. Demographic elements were similarly distributed between treatment groups except gender (Table 1).

### 3.1. Pain Coprimary Endpoint

Thirty-three (50.8%) patients who received TTX and 29 (34.5%) patients who received placebo were responders on the pain coprimary endpoint. The primary analysis of the efficacy data from this study supports a clinically relevant benefit of TTX over placebo on the prespecified pain intensity endpoint with an effect size of 16.2% in favour of TTX (*p* = 0.0460 one-sided *t* test) and a number-needed-to-treat (NNT) value of 6.2. The *p* value of 0.0460 was nominally statistically significant but not at the prespecified two-sided 5% level after the prespecified (Bonferroni Holm) adjustment for the two primary endpoints.

Upon adjustment for the baseline factors of age, opioid level, and pain level (verbal rating scale), the analysis of the pain intensity endpoint showed a greater effect size and nominal statistical significance with an estimated response difference of 23.1% (TTX, placebo), nominal *p* = 0.0127, and NNT of 4.3. Baseline factors of age, opioid level, and pain level (verbal rating scale) were the only cofactors remaining in a standard forward selection logistic regression using an entry threshold of 0.2. The resulting model (primary model) fit for the pain intensity model was very good (Hosmer-Lemeshow *p* = 0.8336).

Conservative sensitivity analyses were conducted to address the issue of potential bias due to dropouts. In the most conservative analysis conducted, upon imputation for the missing data, response for the pain intensity endpoint was decreased by 5.0%, (16.2% versus 11.2%), with *p* value increased by 0.11 (0.16–0.05).

All imputation sensitivity analyses supported efficacy results in the same direction, with somewhat attenuated effect sizes as expected.

### 3.2. Composite Coprimary Endpoint: Pain Plus Quality of Life

Nineteen (29.2%) patients who received TTX and 17 (20.2%) patients who received placebo were responders. However, the proportion of responders to treatment with TTX during EPIP or LPIP was not statistically significant (*p* = 0.2035) with a NNT value of 11.1. The impact of pain on QoL was physical functioning, difference of 4.6%, and *p* = 0.5651 and emotional functioning, difference of 6.9%, and *p* = 0.4011.

### 3.3. Secondary Analyses

All of the planned secondary analyses showed what appeared to be a robust analgesic signal.

#### 3.3.1. Duration of Analgesic Response

**Figure 1:**
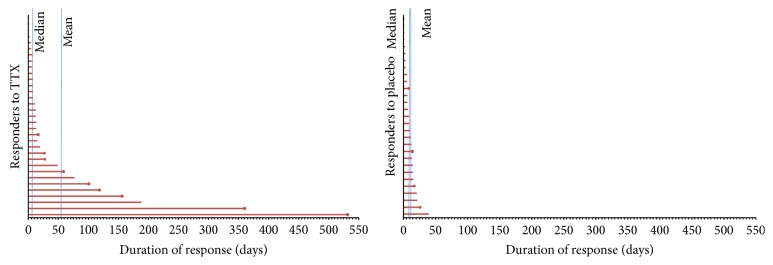
TTX for cancer pain: secondary analyses. Response duration (TTX responders versus placebo responders).

The median duration of response for the pain intensity coprimary endpoint in the responders was 12 days (TTX) versus 8 days (placebo). The means were 56.7 days (TTX) and 9.9 days (placebo) (Figure 1). This median duration of response of approximately 2 weeks for the responders on the pain intensity endpoint may be clinically important, though there was a wide range in duration (1–530 days) in the TTX treatment arm. About one-third of the responders experienced an analgesic effect for extremely long durations. This was not seen in the placebo arm.

#### 3.3.2. Cumulative Proportion of Responders Analyses

**Figure 2:**
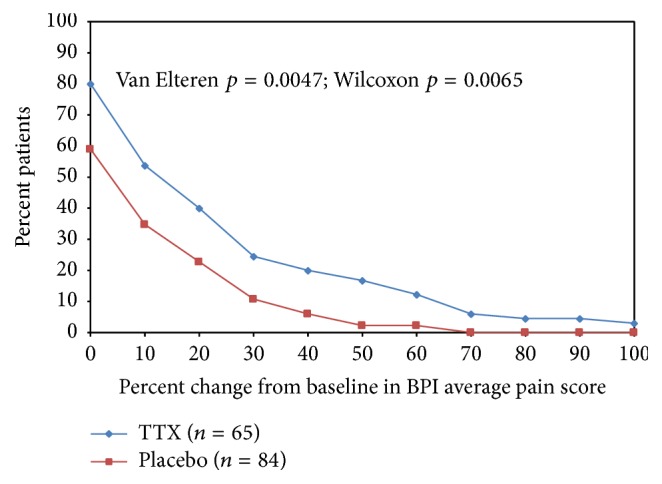
Cancer pain: secondary analyses. % change from baseline average pain (Q5 BPI) Cumulative Proportion of Responders Analyses (ITT) at late postinjection period.

While the primary endpoints use a fall in pain from baseline by at least 30% in the definition of an analgesic response, the cumulative proportion of responders analysis allows a broader comparison of the active and placebo treatment arms by comparing the proportion of patients in both groups who have a reduction by any specific percentage change from baseline of pain (Figure 2). The Cumulative Proportion of Responders Analyses graph shows that the treatment groups are well separated along the entire range of percent change response definition (*x*-axis) during the late postinjection period. Similar graphs were obtained for the early postinjection period as well as for worst pain, most bothersome pain, and opioid use at both early and late postinjection periods (data not shown)  (Van Elteren, *p* = 0.0241, and Wilcoxon, *p* = 0.0511 for EPIP; Van Elteren, *p* = 0.0047, and Wilcoxon, *p* = 0.0065 for LPIP).

#### 3.3.3. Patient Global Impression of Change

**Figure 3:**
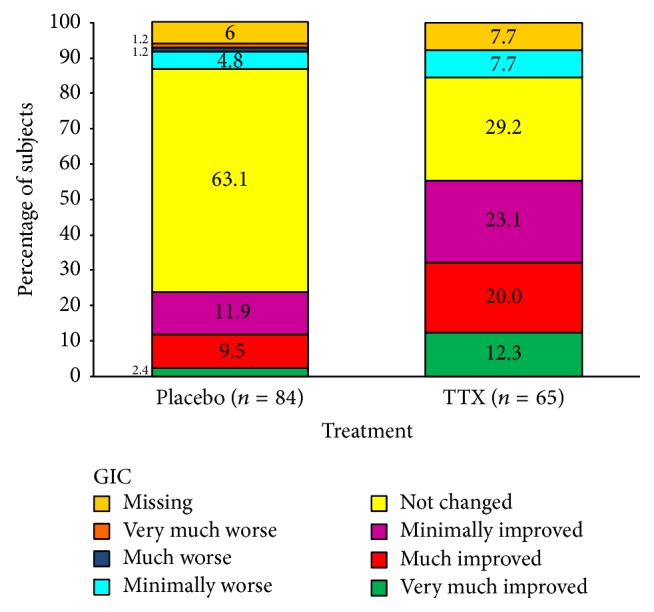
TTX for cancer pain: secondary analyses. Patient global impression of change (GIC) during the late postinjection period.

GIC results at LPIP are presented in Figure 3. Analyses of the patient GIC supported the primary results of a nominally statistically and clinically significant benefit of TTX on pain both at the early postinjection period and at the late postinjection period. The distribution of GIC was different between TTX and placebo at EPIP and LPIP with the majority of patients in the placebo group reporting no change (63.1%) for the most bothersome pain and the majority of patients in the TTX group reporting improvement (55.4%).

When considering a GIC strong response (*very much improved* and* much improved*), at EPIP 30.8% of patients in the TTX group and 8.3% in the placebo group (nominal *p* = 0.0004, chi-square) reported improvement; at LPIP 32.3% versus 11.9% for placebo (nominal *p* = 0.0023, chi-square) (Figure 3); and at EPIP or LPIP 40.0% versus 11.9% for placebo (nominal *p* < 0.0001, chi-square). These differences in GIC strong response translated into clinically significant calculated NNT estimates of 4.4, 4.9, and 3.6 at EPIP, LPIP, and EPIP or LPIP, respectively.

When considering the* minimally improved* response category, the proportion of patients reporting an improvement in the TTX group was 55.4% and in placebo 23.8% at LPIP. The NNT estimates for TTX were also clinically significant: 2.6 for EPIP, 3.2 for LPIP, and 2.7 for EPIP or LPIP. Similar results were obtained in the low baseline opioids subgroup (data not shown).

#### 3.3.4. Additional Analyses: Low Opioid Dose Subgroup

After the completion of the study, it was found that a modest number of patients of the study population, 23 patients, were receiving very high doses of opioids, 500 mg or greater MEDD. These patients had not been a priori excluded from participation in the trial. Of the 126 patients who received less than 500 mg MEDD, 31 (50.8%) in the active treatment arm and 17 (26.2%) in the placebo arm were pain (coprimary endpoint) responders. The difference between the two treatment arms was 24.6%, with a nominal *p* value of 0.0044 (chi-square test). Sensitivity analyses similar to those conducted on the predefined ITT population provided results consistent with these analyses. In the sensitivity analysis for the coprimary pain intensity endpoint in the low baseline opioid subgroup, there was a 20.0% treatment difference in proportion of responders between TTX and placebo (nominal *p* = 0.0149).

For the composite coprimary endpoint, the results for patients receiving less than 500 mg MEDD were similar. The number of responders was significantly higher in the TTX group, 19 (31.1%), compared with the placebo group, 9 (13.8%), for the composite endpoint (nominal *p* = 0.0196).

### 3.4. Adverse Events

Adverse events were generally mild to moderate and transient (Table 2). In the TTX group, all patients (100%) experienced at least 1 TEAE (treatment emergent adverse event) considered related to study drug, while 77 patients (88%) in the placebo group reported at least 1 TEAE related to study drug. The commonest reported adverse events were nausea, dizziness, oral numbness or tingling, and injection site irritation. There were 12 serious adverse events (SAEs). Five of these SAEs, occurring in three patients, were deemed probably related to active treatment: ataxia (2), nystagmus (1), other neurotoxicity (1), and aspiration pneumonia (1). The latter occurred in a patient who had prior extensive head and neck surgery and was presumably at risk for aspiration. No deaths were reported during the course of the study.

## 4. Discussion

At present, the mainstay of pharmacotherapy to manage moderate to severe cancer-related pain generally includes orally administered opioid therapy with or without coanalgesics. However, it is not always effective and is often associated with adverse effects such as constipation, nausea, vomiting, and central nervous system toxicity such as drowsiness, cognitive impairment, and hyperalgesia [1, 10]. Further, cancer patients often have considerable comorbidity which can reduce tolerability of opioids. For a substantial proportion of cancer patients, opioid adverse effects are not manageable. The clinical circumstance where opioid related relief of pain is overshadowed by unmanageable toxicity is referred to as “opioid poorly responsive pain” and is a devastating scenario [2, 11]. Thus, pharmacotherapy remains unsatisfactory for many cancer patients with pain, and for them, there is an urgent need for additional, new nonopioid therapeutic approaches. 

In part, the variability in how patients respond to different analgesics for cancer pain is thought to be related to the wide range of pathophysiologic mechanisms which have been described to be responsible for the development of chronic pain disorders [12]. One described mechanism for chronic pain is the presence of ectopic discharges of voltage-gated sodium channels, which are in abundance in both the peripheral and the central nervous systems. TTX is a selective blocker of Na^+^ channels with a high affinity to Na_V_ 1.7 in the periphery and causes analgesia either by decreasing the propagation of action potentials by Na^+^ channels or by blocking of ectopic discharges associated with chronic pain [3, 4].

The study drug is an injectable formulation of highly purified TTX extracted from the puffer fish (Fugu). Data from our group involving dose tolerance studies in healthy human patients have demonstrated that single doses up to 45 *μ*g or multiple doses up to 36 *μ*g q.i.d. were generally tolerated both as an acute single intramuscular (i.m.) injection and multiple i.m. doses for 4 or 7 days. Administration of TTX by subcutaneous injection elicits a pharmacological effect similar to that achieved when it is administered by intramuscular injection [6, 7].

A Phase IIa open-label study found that 30 *μ*g b.i.d. dose of TTX administered intramuscularly for 4 days appeared to be safe and efficacious in cancer patients whose pain was not relieved with standard therapy that included strong opioids and adjuvant analgesics [6]. The role of TTX in cancer-related pain was subsequently assessed in a larger Phase II randomized, multicentre, double-blind, placebo-controlled trial of subcutaneous TTX in patients with moderate or severe cancer pain unrelieved by best available treatment [7]. The design called for TTX to be administered subcutaneously over Days 1–4 with a period of observation to Day 15 or longer. The primary endpoint was the proportion of responders defined as patients who had a ≥30% decrease in mean pain intensity during the EPIP (Days 5–8) or LPIP (Days 9–15). The mean opioid dose (expressed as MEDD) during the same period was less than 125% of the mean opioid dose during the baseline period.

In that Phase IIa study, 82 patients were randomized, and results for 77 patients were used for analysis. A futility interim analysis was conducted and the results showed that there was a nonstatistically significant trend toward more responders in the active treatment arm based on the primary endpoint (pain intensity difference) and the DMC recommended to stop the study for futility. However, analysis of secondary endpoints and an exploratory post hoc analysis suggested that a robust analgesic effect might have been shown if a composite endpoint had been used, for example, an evaluation that combines pain outcome (pain intensity) and QoL. This retrospective analysis suggested that TTX may potentially relieve moderate or severe, treatment resistant cancer pain in a large proportion of patients, and for prolonged periods following treatment, but further study was recommended using a primary composite endpoint.

The current study, TEC-006, adds to these earlier, smaller trials and provides further evidence for the safety and efficacy of TTX at the prescribed dose and schedule. The TEC-006 study was intended to be a pivotal trial that is to bring the drug to market in Canada. Firm statistical conclusions are however difficult since the TEC-006 study was stopped prematurely well before the planned sample size was reached; it is an underpowered study. The study demonstrates a treatment difference across endpoints and, adjusting for baseline imbalances, the treatment difference was consistent with a clinically meaningful analgesic signal but not reaching statistical significance in the prespecified primary analyses. We interpret these results to be most consistent with the study being underpowered (i.e., a type II error) as the main cause. Even with this smaller patient cohort, in a group of heavily pretreated patients and with advanced disease, the pain coprimary endpoint fell just short of statistical significance, with a clinically meaningful NNT between 4 and 6. All secondary analyses showed a similar analgesic effect: duration of analgesia, patient global impression of change, the cumulative proportion of responders, and the low opioid subgroup analysis. Strikingly, a large group of patients experienced a prolonged analgesic effect lasting weeks or even months. There was no underlying mechanism of pain – somatic, visceral, neuropathic, or mixed pain – which showed a greater or lesser likelihood of such a response.

The study design did not exclude patients receiving very high dose opioids (500 mg or greater MEDD) from enrollment in the trial. The literature shows that patients on very high doses of opioid are likely to represent a group of patients who are difficult to manage and have unpredictable treatment outcomes [13]. Some analgesic clinical trials have attempted to mitigate this potential confounding variable by a priori exclusion of patients who are receiving high baseline doses of opioids with the understanding that this group of patients has poorly responsive pain and a poorer pain outcome. Taking these collective experiences into consideration, analyses of the TEC-006 data were also performed in the subgroup of patients receiving lower doses of opioids (less than 500 MEDD). Although the primary pain endpoint results in this subgroup were highly statistically significant, the analysis was unfortunately not prespecified.

## 5. Conclusions

While underpowered, this multicentre, randomized, double-blind, placebo-controlled, parallel-design trial suggests a clinically important analgesic effect (NNT about 4–6) in a cohort of heavily pretreated patients with advanced illness and otherwise with poorly controlled moderate to severe cancer pain. The pain reduction primary endpoint was nominally statistically significant; however, because of the statistical penalties for multiplicity, the study could not be considered statistically positive. There was further indication of an analgesic signal: with a convergence of positive findings within the secondary and post hoc exploratory analyses. Finally, the results of this trial support the hypothesis that patients requiring high dosages of opioids, for example, 500 mg MEDD or greater, are poor candidates for TTX pain therapy.

We conclude that TTX demonstrates a favourable benefit-risk profile in the treatment of uncontrolled moderate to severe cancer-related pain and may play an important role in addressing a major unmet medical and societal need. The promising results suggesting prolonged analgesia lasting weeks or even months following four days of administration need to be confirmed, for example, through a sufficiently powered Phase III clinical trial.

## Figures and Tables

**Table 1:** Patient demographics.

	TTX (*N* = 77)	Placebo (*N* = 88)
Age (years)		
*N*	77	88
Mean (SD)	55.5 (11.2)	54.8 (11.5)
Gender, *n* (%)		
*N*	77	88
Male	27 (35.1)	44 (50.0)
Female	50 (64.9)	44 (50.0)
Race, *n* (%)		
*N*	77	88
Caucasian	72 (93.5)	84 (95.5)
Asian	1 (1.3)	1 (1.1)
African American	1 (1.3)	0 (0.0)
Other	3 (3.9)	3 (3.4)
Height (cm)		
*N*	71	78
Mean (SD)	165.8 (9.6)	168.4 (8.9)
Weight (kg)		
*N*	72	77
Mean (SD)	73.4 (18.3)	78.2 (19.1)
BMI (kg/m^2^)		
*N*	70	76
Mean (SD)	26.7 (5.4)	27.4 (5.9)

BMI: body mass index; SD: standard deviation.

**Table 2:** Most common adverse events.

Most common AEs	TTX	Placebo
Nausea	68% (10% severe)	23% (2% severe)
Dizziness	61% (4% severe)	18% (0% severe)
Oral hypoesthesia	61% (0% severe)	9% (0% severe)
Hypoesthesia	48% (0% severe)	10% (0% severe)
Oral paresthesia	44% (0% severe)	2% (0% severe)
Vomiting	34% (3% severe)	8% (0% severe)
Injection site irritation	52% (6% severe)	53% (7% severe)
Serious adverse events (*n* = 12)	6 patients	3 patients
SAEs related to TTX (after unblinding)	5 events from 3 subjects: ataxia (2), neurotoxicity (1), nystagmus (1), and aspiration pneumonia (1)	

Statistical analyses of safety endpoints were descriptive only.
